# Shear Wave Elastography in the Diagnosis of Peripheral Neuropathy in Patients With Chronic Kidney Disease Stage 5

**DOI:** 10.3389/fendo.2022.899822

**Published:** 2022-06-23

**Authors:** Xuan Li, Haoqi Sun, Zhaoguang Zhang, Jing Liu, Huiying Xu, Lin Ma, Haibo Zhang, Jialin Li, Qian Luo, Xiangming Wang, Min Guo, Zhentao Guo, Xuexun Chen

**Affiliations:** ^1^Department of Nephrology, Affiliated Hospital of Weifang Medical University, Weifang, China; ^2^Department of Ultrasound, Affiliated Hospital of Weifang Medical University, Weifang, China; ^3^Department of Electrophysiology, Affiliated Hospital of Weifang Medical University, Weifang, China

**Keywords:** chronic kidney disease, peripheral neuropathy, cutoff value, tibial nerve, elasticity imaging techniques

## Abstract

**Objective:**

To observe the feasibility of shear wave elastography (SWE) in the diagnosis of peripheral neuropathy in patients undergoing hemodialysis [chronic kidney disease stage 5 dialysis (CKD5D)].

**Methods:**

Forty patients with CKD5D were divided into a uremic peripheral neuropathy (UPN) group (n = 25) and a non-UPN group (n = 15) according to the results of a neuro-electrophysiological examination. Sixteen healthy control subjects were also enrolled in this study. Two-dimensional ultrasound examination was conducted, and SWE was then performed to measure Young’s modulus of the tibial nerve. The left and right diameters (D1), anterior and posterior diameters (D2), perimeter (C), cross-sectional area (CSA), and Young’s modulus (E) were measured three times at the same non-entrapment site. The average values were recorded and calculated. The following evaluation indices were also analyzed: sensitivity, specificity, positive predictive value (PPV), negative predictive value (NPV), and area under the receiver operating characteristic curve (AUC).

**Results:**

D1, D2, C, and CSA were not significantly different among the three groups (P > 0.05). However, the difference in the E value among the three groups was statistically significant (P < 0.05). The AUC was 0.889 based on the E value. Using a tibial nerve E value of 48.35 kPa as the cutoff value, the sensitivity, specificity, PPV, and NPV were 86.0%, 84.0%, 81.1%, and 88.1%, respectively.

**Conclusions:**

SWE is useful for the diagnosis of peripheral neuropathy in patients with CKD5D. Young’s modulus of 48.35 kPa for the tibial nerve is the optimal cutoff value and has the best diagnostic efficiency for peripheral neuropathy in CKD5D patients.

## Introduction

Chronic kidney disease (CKD) is a global health problem that reduces quality of life and disrupts economic development. One of the most common neurological complications of CKD, especially CKD stage 5 dialysis (CKD5D), is peripheral neuropathy, which affects approximately 60% to 90% of patients with CKD (1). Uremic peripheral neuropathy (UPN) has characteristic symptoms or can be detected by clinical examinations. The most common and earliest symptoms are mainly sensory dysfunctions, such as pain and paresthesia; these are followed by limb weakness and atrophy (2). However, some patients with impaired nerve function exhibit no clinical symptoms (3, 4). Thus, a nerve conduction study (NCS) is usually performed to evaluate the function of peripheral nerves. However, the nerve conduction is not sensitive enough to detect peripheral neuropathy, especially in asymptomatic patients (5).

Ultrasound (US) elastography has become an important tool for the evaluation of nerve stiffness. This procedure takes less time to perform and causes less discomfort to patients. US examinations are used to assess the elasticity of the nerve in patients undergoing hemodialysis by measuring the nerve cross-sectional area (CSA) (6, 7). Two-dimensional shear wave elastography (2D-SWE) is a new complementary method to NCS that has been widely applied for the detection of diabetic peripheral neuropathy in recent studies (8, 9). It can reveal minor peripheral nerve lesions that cannot be detected by electrophysiology (8) and has higher sensitivity and specificity than US, which is mainly based on CSA measurement (6). Although research has revealed changes in nerve elasticity in patients undergoing hemodialysis, few studies have used 2D-SWE for the detection of peripheral nerve damage in these patients. Thus, the diagnostic performance of 2D-SWE in patients undergoing hemodialysis was evaluated in the present study.

## Materials and Methods

### Ethics and Consent

This study was approved by the ethics committee of the Affiliated Hospital of Weifang Medical University. All participants provided written informed consent.

### Participants

Forty patients (15 women, 25 men) undergoing hemodialysis were recruited from the Affiliated Hospital of Weifang Medical College dialysis center. All patients underwent electrophysiological tests. Sixteen healthy volunteers with no clinical signs or symptoms were enrolled as the control group. All participants underwent US and 2D-SWE examinations. The following basic data were collected for all participants: sex, age, hemoglobin (Hgb), hematocrit (HCT), albumin (Alb), total protein (TP), blood lipid indices, blood urea nitrogen (BUN), serum creatinine (Scr), parathyroid hormone (PTH), beta-2 microglobulin, blood sodium, blood potassium, blood calcium, blood phosphorus, and duration of dialysis. The inclusion criteria were treatment with hemodialysis in our dialysis center and good cognitive function and communication skills; and the age ranged from 18 to 78 years. The exclusion criteria were polyneuropathy caused by diabetes, hereditary factors, alcohol intake, metabolic factors, inflammatory factors, a malignant tumor, or toxic factors; skin lesions or swelling of the ankle; leg or ankle fractures; and damage to the liver, brain, heart, lung, or other important organs.

### Diagnosis of UPN

The diagnosis of UPN is based on symptoms and clinical examination findings (10). Patients may have one or more of the following clinical symptoms: paresthesia, restless leg syndrome, increased pain sensation, impaired deep tendon reflexes, imbalance, numbness, and atrophy of the lower limbs (11–15). An NCS is currently regarded as the most effective method to diagnose peripheral neuropathy. Thus, it is used as the gold standard for the diagnosis of UPN (11, 12). In the present study, one neurologist with 10 years of experience in NCSs performed an NCS for all patients. The patients were grouped according to the results.

### Electrodiagnostic Studies

Electromyography was performed using a conventional procedure on a standard system (Nicolet EDX; Natus Medical, Middleton, WI, USA). All examinations were performed in the same room at an ambient temperature of 25°C. They were begun 1 hour after a hemodialysis session because normalization of nerve excitability parameters can occur after hemodialysis (16, 17). An NCS of the bilateral tibial nerves was performed in every patient. We obtained all relevant data including latencies, amplitudes, and conduction velocity. The case definition for UPN was based on the results of electromyography, which is the gold standard method (18).

### Patient Positioning

All patients were placed in the supine position during the examination. Their ankles were relaxed. To prevent the effect of ankle soft tissue pressure, ankle movement was avoided during the examination.

### US and SWE Measurements

The whole study population underwent SWE and US examinations by Sonographer 1 (Zhaoguang Zhang, with 10 years of experience in US and 3 years of experience in elastography). Six of the participants were randomly selected for a second SWE examination by Sonographer 1 after 24 hours and they were performed by Sonographer 2 (Qian Luo, with 8 years of experience in US) by SWE. Both Sonographers were blinded to the participants’ information, including their clinical history, previous examination findings, and NCS results. All SWE examinations were completed within 1 week after the NCS.

The examinations were performed using a device with an L2-9-D transducer (LOGIQ E20; GE Healthcare, Chicago, IL, USA). The transducer was gently placed onto the skin surface. Care was taken to perform the examination with light contact using ample coupling gel. The tibial nerve was scanned upward from the medial malleolus to examine the cross section of the tibial nerve, determine the tibial nerve boundary, observe the internal structure of the tibial nerve, and assess the echo of the nerve bundle. The tibial nerve CSA was measured 4 cm above the medial malleolus. The transducer was then rotated 90° to view the longitudinal imaging plane. The depth of the image was adjusted to find the tibial nerve, and SWE was then performed. Three iterative measurements were obtained at 2-min intervals. The validated measurements of each tibial nerve were taken by each sonographer. Young’s modulus (E) was expressed in kilopascals (kPa).

### Statistical Analysi*s*

SPSS version 17.0 software (SPSS Inc., Chicago, IL, USA) was used for the statistical analysis. Categorical variables are presented as percentage. Continuous data are presented as mean ± standard deviation or median and interquartile range. An independent t-test and One-way analysis of variance were performed for statistical comparisons, and then the LSD test was used for multiple comparisons. Non-parameter test was used for non-normal distribution data. Kruskal-Wallis test and Mann-Whitney *U* test were performed to compare variables. The best cut-off value of the nerve E value was obtained by plotting receiver operating characteristic curve. The interclass correlation coefficient (ICC) was used to evaluate the intra- and inter-observer reliability. Statistical significance was accepted at P <0.05.

## Results

### Clinical Baseline Characteristics

Clinical baseline data were collected from 56 participants (40 patients undergoing hemodialysis and 16 healthy control individuals). These baseline characteristics are displayed in **Table 1**. The statistical analysis showed no significant differences in age, HCT, Alb, TP, cholesterol, low-density lipoprotein cholesterol, or calcium among the three groups (P > 0.05). BUN and Scr were significantly higher in the CKD5D patients than in the controls (P < 0.05). CKD5D patients had lower Hgb than the controls (P < 0.05). The UPN group had more severe hyponatremia and hyperkalemia than the non-UPN and the control groups (P < 0.05). Triglycerides in the UPN group were higher than in the control group (P < 0.05). However, there were no significant differences in Hgb, BUN, Scr, PTH, phosphorus, triglycerides and beta-2 microglobulin between the UPN group and non-UPN group (P > 0.05). The UPN group had a longer duration of dialysis than the non-UPN group (P < 0.05).

**Table 1:** The general characteristics of three groups.

	UPN (n=25)	Non-UPN(n=15)	Healthy control(n=16)	*P* value
Male [n(%)]	16(64.0%)	9(60.0%)	8(50.0%)	
Age(years)	56.21 ± 11.85	50.73 ± 16.21	55.25 ± 10.15	0.414
Years on dialysis	3.25(2.00,6.13)^c^	1.00(0.16,2.50)	–	0.009
Hgb(g/l)	112.04 ± 23.37^a^	104.13 ± 22.64^b^	126.88 ± 14.95	0.013
HCT(L/L)	0.34 ± 0.07	0.32 ± 0.08	0.38 ± 0.05	0.061
Alb(g/l)	39.07 ± 7.61	38.31 ± 5.27	38.49 ± 6.09	0.932
TP(g/l)	65.15 ± 7.48	61.43 ± 8.37	63.88 ± 7.65	0.355
Cholesterol (mmol/l)	4.23 ± 1.31	4.14 ± 1.32	4.62 ± 0.95	0.524
Triglycerides(mmol/l)	1.70(1.29,1.90)^a^	1.29(0.76,1.92)	1.09(0.83,1.28)	0.021
LDL-cholesterol (mmol/l)	2.45 ± 1.11	2.12 ± 1.02	2.30 ± 1.00	0.658
Sodium (mmol/l)	138.97 ± 4.23^a^^,c^	141.71 ± 3.60	141.77 ± 2.15	0.023
Potassium (mmol/l)	5.22 ± 1.37^a^^,c^	4.55 ± 1.21	4.05 ± 0.44	0.007
Calcium(mmol/l)	2.19 ± 0.34,	2.20 ± 0.34	2.24 ± 0.12	0.860
Phosphorus(mmol/l)	2.17 ± 0.81	2.18 ± 0.46	–	0.949
BUN(mmol/l)	27.21 ± 10.34^a^	26.65 ± 8.31^b^	4.72 ± 1.02	< 0.001
Scr(umol/l)	892.68 ± 285.23^a^,	972.06 ± 218.45^b^	0.25 ± 14.61	< 0.001
PTH(pmol/l)	143.00(65.90,410.80)	191.20(109.00,320.00)	–	0.544
Beta2 microglobulin (mg/l)	22.53 ± 3.83	22.68 ± 3.65	–	0.934

^a,b^Compared with Healthy control, ^c^Compared with non-UPN nephropathy, P < 0.05; P < 0.05 is considered statistically significant. Hgb, hemoglobin; HCT, hematocrit; Alb, albumin; TP, the total protein; LDL, cholesterol low density lipoprotein-cholesterol; BUN, blood urea nitrogen; Scr, serum creatinine; PTH, parathyroid hormone.

### US Features of Tibial Nerve

**Figure 1:**
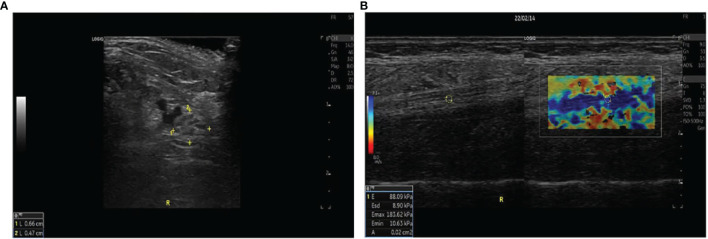
**(A)** The left and right diameters, anterior and posterior diameters, perimeter, and CSA of the tibial nerve were measured 4 cm proximal to the medial level in a 57-year-old woman with UPN. **(B)** Two split US and SWE images were obtained at the same longitudinal level in a 39-year-old man with UPN. Quantitative SWE measurements showed that the mean nerve stiffness was 88.09 kPa. R, Right.

**Figure 2:**
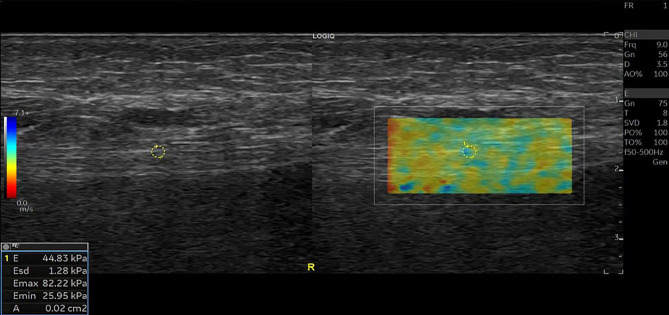
SWE image of the tibial nerve in a 52-year-old man with non-UPN. Quantitative SWE measurements showed that the mean nerve stiffness was 44.83 kPa. R, Right.

**Figure 3:**
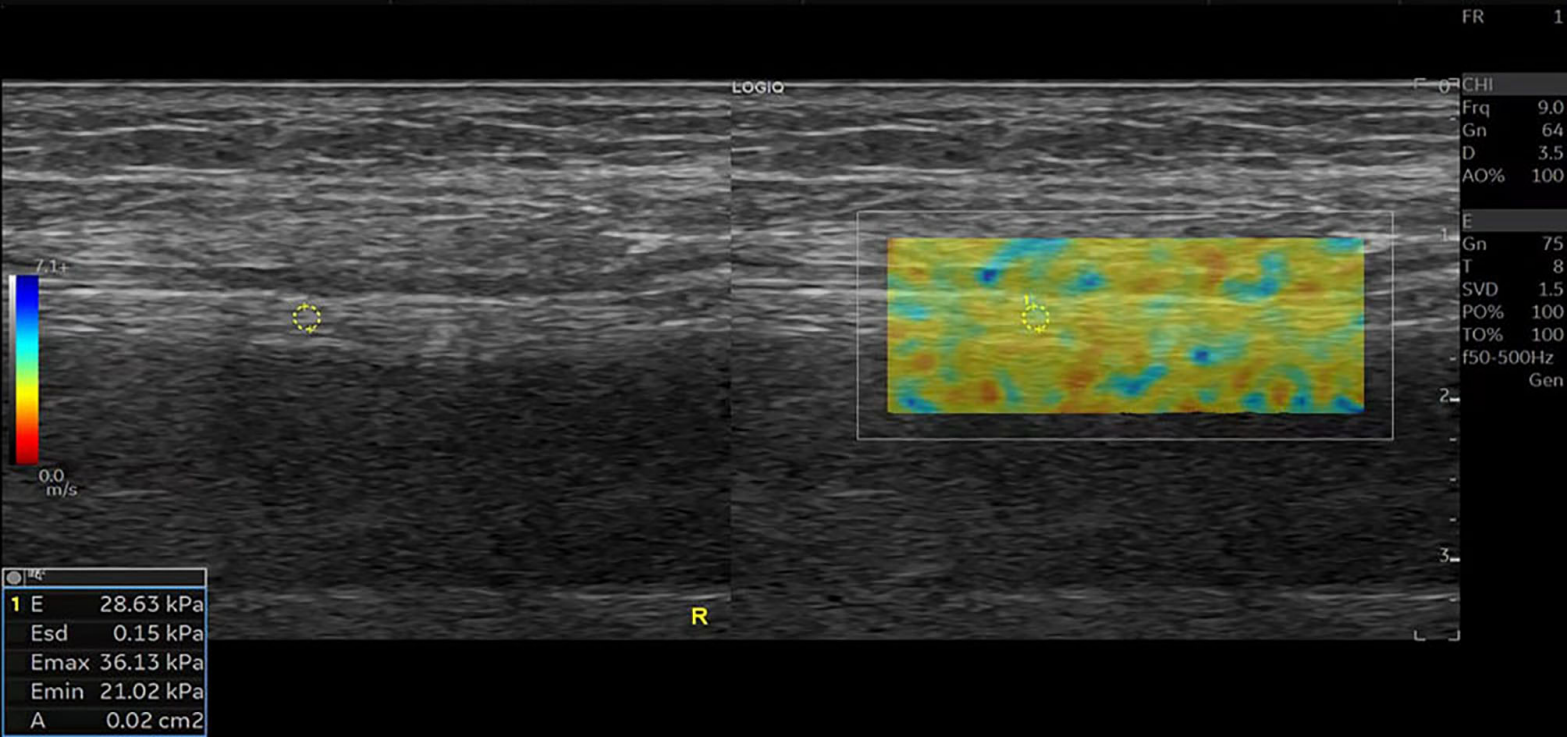
SWE image of the tibial nerve in a 56-year-old healthy man. Quantitative SWE measurements showed that the mean nerve stiffness was 28.63 kPa. R, Right.

A total of 80 ankles of 40 CKD5D patients and 32 ankles of 16 healthy controls were enrolled in our study. The measurement indices of the tibial nerve in all three groups are displayed in **Table 2**. There was no significant difference in the left and right diameters, anterior and posterior diameters, perimeter, or CSA of the tibial nerve among the three groups (P > 0.05). The stiffness of the tibial nerve was measured with 2D-SWE and the shear elasticity index was displayed as Young’s modulus(E). The stiffness of the tibial nerve in the UPN group (**Figure 1**) was significantly different from that in the non-UPN group and control group (P < 0.05) and was significantly different between the non-UPN group (**Figure 2**) and control group (P < 0.05) (**Figure 3**).

**Table 2:** The values in the tibial nerve for each group.

	UPN (n=50 ankles)	Non-UPN (n=30 ankles)	Healthy control (n=32 ankles)	*P* value
D1(mm)	5.56 ± 0.86	5.73 ± 0.66	5.67 ± 0.56	0.556
D2(mm)	3.87 ± 0.76	4.05 ± 0.64	3.94 ± 0.56	0.522
C(cm)	1.79 ± 0.17	1.82 ± 0.16	1.75 ± 0.13	0.244
CSA(cm^2^)	0.22 ± 0.05	0.22 ± 0.04	0.20 ± 0.04	0.417
E(kPa)	65.16 ± 17.82^a,c^	48.44 ± 10.24^b^	33.63 ± 6.43	< 0.001

^a,b^Compared with Healthy control, ^c^Compared with non-UPN nephropathy, P < 0.05; P < 0.05 is considered statistically significant. D1, left and right diameters; D2, anterior and posterior diameters; C, perimeter; CSA, cross-sectional area; E, Young’s modulus values.

### Determination of Optimal Cut-Off Value of E for Diagnosis of UPN Based on US and SWE Measurements

**Figure 4:**
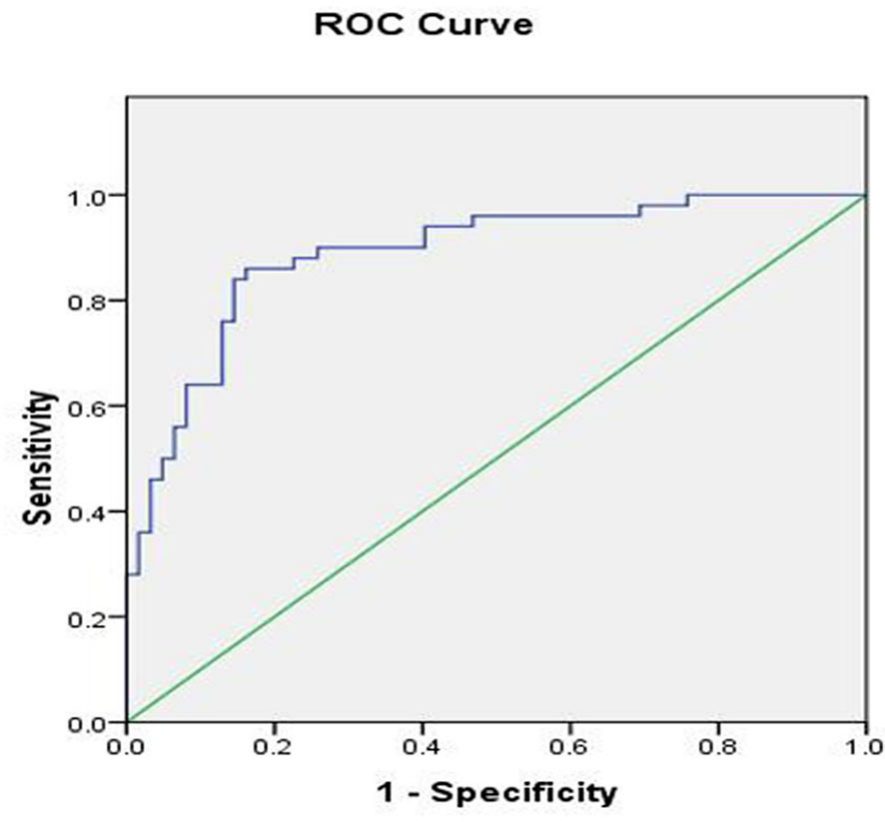
Receiver operating characteristic curve based on SWE measurement values.

To determine the best cut-off value of E, we performed a receiver operating characteristic curve analysis based on the E value of the tibial nerve as shown in **Figure 4**. The optimal cutoff value of E for diagnosing UPN was 48.35 kPa. The sensitivities, specificities, positive predictive values, and negative predictive values are summarized in **Table 3**.

**Table 3:** The efficiency of the cut-off values in diagnosing UPN.

	Cutoff	Sensitivity,%	Specificity,%	PPV,%	NPV,%	Youden index	AUC
E(kPa)	48.35	86.0(43/50)	84.0 (52/62)	81.1 (43/53)	88.1(52/59)	0.699	0.889

E, Young’s modulus values; AUC, area under the receive operating characteristic curve; PPV, positive predictive value; NPV, negative predictive value.

### Inter- and Intra-Observer Consistency Analysis

The ICC of the intra-observe was 0.950(95% CI 0.350, 0.990). The inter-observe ICC was 0.931(95% CI 0.786, 0.979). SWE showed very good inter- and intra-observer consistency.

## Discussion

Peripheral neurological complications are very common in patients with CKD, with an estimated prevalence of up to 90% in the CKD5D population (19). Such complications present as a slowly progressive sensorimotor neuropathy. The clinical manifestations may include pain, paresthesia, numbness in the distal lower limbs, or loss of sensitivity. Peripheral neuropathy results from a variety of mechanisms. Diabetic neuropathy is one neurological manifestation of end-stage renal disease (ESRD) (12). The mechanisms of diabetic neuropathy are well known and widely reported. With respect to CKD-induced neuropathy, many substrates have been investigated as potential uremic neurotoxins. The retention of neurotoxic solutes with a molecular weight of 300 to 2000 Da, including PTH and beta-2 microglobulin, has been discussed as a cause of UPN because such solutes are slowly dialyzable (20). However, urea, creatine, and uric acid have shown no evidence of causality. Many nerve excitability studies in uremic neuropathy have provided evidence that hyperkalemia is related to nerve dysfunction and contributes to the development of neuropathy (21). Similarly, it showed more sever hyperkalemia in the UPN group than the non-UPN and the control groups in our study. The UPN group had lower sodium than the non-UPN and the control groups. Previous studies showed that hyponatremia may be related to the central nervous system toxicity *via* multiple pathways (22). It was uncertain whether lower serum sodium levels promoted injury to the peripheral neuropathy, which needed further research. In the present study, the sex distribution among the three groups was not revealed significant difference. However, Said et al. (15) found that uremic neuropathy is more common in male than in female patients. Additionally, Hojs-Fabjan et al. (23) found that polyneuropathy was associated with the patient’s age and duration of dialysis treatment. In our study, the years of dialysis were longer in the UPN group than in the non-UPN group, which is consistent with the study by Hojs-Fabjan et al. (23). No significant difference in age was found among the three groups. The BUN and Scr concentrations were significantly higher in the CKD5D patients than in the healthy controls. Abnormal lipoprotein profile has been reported in CKD5D patients (24). Triglycerides were much lower in the UPN group than in the control group. However, no significant differences were in Hgb, HCT, Alb, TP, cholesterol, low-density lipoprotein cholesterol, calcium, phosphorus, Scr, BUN, PTH, or beta-2 microglobulin between the UPN group and non-UPN group.

The gold standard method for the diagnosis of peripheral neuropathy is an NCS, which is time-consuming and invasive. High-resolution US has recently become more widely used in the detection of neuropathy because of its low cost, noninvasiveness, and ability to depict the location and range of the lesion. Our study showed no significant difference in the anterior and posterior diameters, left and right diameters, perimeter, or CSA of the tibial nerve among the three groups, indicating that nerve damage has little effect on the morphological characteristics of nerves. Consistent with the findings reported by Dikici et al. (8), the morphological changes were not obvious when the nerve was uncharacteristically damaged.

Through the measurement of Young’s modulus, SWE can quantitatively reflect the elasticity of tissue. Harder tissue has a greater Young’s modulus. This measurement index has been widely applied in liver disease (25), thyroid disease (26), diabetic peripheral neuropathy (8), and other conditions. However, few studies have been performed to assess the peripheral nerve elasticity in CKD5D patients. In this study, we evaluated the tibial nerve stiffness in such patients. The inter-observer and intra-observer reproducibility of SWE were excellent. The E value was statistically significant among the three groups. These findings suggest that more severe lesions are associated with greater nerve stiffness. Our group is currently performing animal studies to elucidate the mechanism of this finding. We demonstrated that the E value had high accuracy for identifying UPN. When 48.35 kPa was taken as the optimal value, the sensitivity was high (86.0%), specificity was high (84.0%), Youden’s index was 0.699, and area under the curve was 0.889. Consistent with previous studies, SWE had better sensitivity and specificity than the CSA for diagnosis of neuropathy (27).

This study had some limitations. First, multiple neuropathies may occur in patients with ESRD undergoing hemodialysis; however, we only evaluated the tibial nerve at one point. More nerves and more measurement points of one nerve are needed. Second, we only evaluated patients at a single hemodialysis center, and these patients represent only a fraction of all patients with ESRD. These findings must be validated by large-sample multicenter prospective studies. Finally, we did not investigate the mechanism underlying the increased nerve stiffness in patients with ESRD. The cause of the change in nerve stiffness with time after dialysis is not clear. Therefore, studies in which different time points after dialysis are examined are needed to observe the changes in nerve stiffness.

In conclusion, SWE is useful for the diagnosis of peripheral neuropathy in CKD5D patients. Young’s modulus of 48.35 kPa of the tibial nerve was taken as the optimal cutoff value for the diagnosis of peripheral neuropathy in CKD5D patients, with a sensitivity of 86.0%, specificity of 84.0%, and area under the curve of 0.889.

## Data Availability Statement

The original contributions presented in the study are included in the article/**Supplementary Material**. Further inquiries can be directed to the corresponding authors.

## Ethics Statement

The studies involving human participants were reviewed and approved by Affiliated Hospital of Weifang Medical University. The patients/participants provided their written informed consent to participate in this study.

## Author Contributions

XL and HS: writing-original draft preparation, formal analysis and visualization. ZZ, JL, and QL: methodology and investigation. XC and ZG: conceptualization, methodology, funding acquisition and writing-review and editing. HX, LM, HZ, and JLL: resources and data curation. XW and MG: supervision and funding acquisition. All authors have read and agreed to the published version of the manuscript.

## Funding

This work was supported by Weifang Key Laboratory of Integrated Traditional Chinese and Western Medicine for Chronic renal Failure; the National Science Foundation of Shandong Province (ZR2021MH394 to XW), Medical and health Science and Technology Development Project of Shandong Province (2017WS172 to XC; 2017WS890 to ZZ), Weifang Soft Science Research Plan (2021RKX047 to XL; 2019RKX088 to XC).

## Conflict of Interest

The authors declare that the research was conducted in the absence of any commercial or financial relationships that could be construed as a potential conflict of interest.

## Publisher’s Note

All claims expressed in this article are solely those of the authors and do not necessarily represent those of their affiliated organizations, or those of the publisher, the editors and the reviewers. Any product that may be evaluated in this article, or claim that may be made by its manufacturer, is not guaranteed or endorsed by the publisher.
